# Effect of Air-Polishing on Titanium Surfaces, Biofilm Removal, and Biocompatibility: A Pilot Study

**DOI:** 10.1155/2015/491047

**Published:** 2015-12-31

**Authors:** Vincent Bennani, Linda Hwang, Andrew Tawse-Smith, George J. Dias, Richard D. Cannon

**Affiliations:** ^1^Sir John Walsh Research Institute, University of Otago, Dunedin 9016, New Zealand; ^2^Department of Anatomy, University of Otago, Dunedin 9016, New Zealand

## Abstract

*Purpose.* The aims of this* in vitro* study were to evaluate morphological changes induced by glycine powder air-polishing on titanium surfaces, biofilm removal, and biocompatibility.* Material and Methods.* Titanium grade IV discs were allocated into two groups: (1) discs without biofilm and (2) discs for* Streptococcus mutans* biofilm formation. Discs in each group were further subdivided into (a) no treatment and (b) air-polishing treatment with glycine powder. Discs were characterized by scanning electron microscopy (SEM), electron-dispersive spectroscopy (EDS), and confocal microscopy. Bacterial biofilms were quantified using a crystal violet dye-binding assay. Biocompatibility was evaluated by measuring the coverage and viability of L929 fibroblast cells cultured on the discs.* Results.* Air-polishing increased the roughness of treated discs (*P* < 0.05). EDS analysis did not show significant differences in the chemical composition of treated and nontreated discs. The amount of residual biofilm on treated discs was 8.6-fold lower than untreated controls (*P* < 0.05). Coverage of treated discs by fibroblasts was half that of untreated discs (*P* < 0.05) although both groups had the same cell viability.* Conclusions.* Air-polishing removed a significant amount of biofilm from titanium surfaces. The “polishing” was accompanied by increased surface roughness, but there were no changes in chemical and elemental compositions, nor the biocompatibility.

## 1. Introduction

Peri-implantitis is an inflammatory disease that affects both the mucosa and the supporting bone around implants. Signs of peri-implantitis include crestal bone loss, deep peri-implant pocketing, bleeding on probing, suppuration, and, in advanced cases, implant mobility [1]. In contrast, peri-implant mucositis is an inflammatory condition that is localized to peri-implant soft tissue without peri-implant bone loss [2].

Microorganisms play a key role in the initiation and development of peri-implant diseases [1]. The microbiota associated with peri-implant disease is complex and often shows very similar microbial composition to that involved in gingivitis and periodontitis [2]. However, it is reported that high proportions of cocci, motile bacilli, and spirochetes are often associated with peri-implant mucositis, whilst high numbers of certain bacterial species including* Porphyromonas gingivalis, Prevotella intermedia, Fusobacterium nucleatum, *and* Treponema denticola* and streptococcal species, including* Streptococcus mutans*, are reported to be associated with peri-implantitis [2–5]. According to a recent study,* Fusobacterium* and streptococcal species were found to be predominant in both peri-implantitis and periodontitis sites, whilst* Parvimonas micra* was only present at peri-implantitis sites [6]. Although* S. mutans* is not usually associated with active peri-implantitis, it is commonly found in the oral cavity and considered as one of the early colonizers of oral surfaces. The bacteria produce extracellular polysaccharide in response to dietary sucrose that firmly attaches the cells to surfaces and contributes to the biofilm matrix.

The incidence of peri-implantitis is approximately 20% of implant patients after 10 years of placement. The prevalence of peri-implant mucositis is even higher. Almost 80% of implant patients in a long-term follow-up (9 to 14 years) reported to have this condition [7]. However, the incidence can vary depending on the criteria used to define the condition, the evaluation period, and treatment protocols. Nevertheless, with the increasing popularity of implants, it is reasonable to predict that the incidence of peri-implant disease will increase. It is also evident that peri-implantitis is the most common cause of late implant failure [8]. The prevalence of peri-implant diseases (peri-implant mucositis) and severity of consequences indicate that there should be effective methods for their prevention and treatment. Presently, there are various treatment modalities available for peri-implant diseases including mechanical methods, chemical methods, and laser applications [9]. Traditionally, mechanical options for peri-implant disease include scaling, implantoplasty, and air-polishing therapy. Chemical methods include subgingival irrigation with antiseptics or antibiotic application [9–12]. The underlying principle in any type of treatment is to reduce bacterial load in peri-implant sites and to achieve peri-implant mucosal health [13, 14]. However, current evidence for the efficacy of each type of treatment is weak and limited, and the superiority of any modality over the others is unknown [1, 14, 15].

In air-polishing therapy, the biofilm is removed by abrasion at the implant surface caused by low-abrasive powders, water, and pressurized air emitted from the device. A range of abrasive powders are available including sodium bicarbonate, amino-acid glycine salt, aluminium trioxide, and calcium carbonate [16]. According to* in vitro* and* in vivo* studies, glycine powders are less abrasive than sodium bicarbonate powders; they are safe to use and effective in biofilm removal [16, 17]. A recent* in vivo* study suggests that glycine powders may inhibit, to some degree, bacterial recolonization of implant surfaces over a 24 h period [18].

The results of many studies, however, are confounded by not controlling the device settings (air pressure and water flow rate) and treatment protocols (distance of the instrument to the surface, instrumentation time, and angulation of the central beam). In addition, further research is needed to clarify any biocompatibility issues of implant surfaces with host tissues that may arise after the air-polishing treatment using glycine powder. Hence, the aims of this research were to evaluate the influence of air-polishing therapy on titanium surface morphology and its effect on biofilm removal and biocompatibility.

The null hypotheses of this study are that (1) glycine powder air-polishing of titanium surfaces has no effect on surface morphology and (2) the powder air-polishing of titanium surfaces has no effect on biofilm removal and biocompatibility.

## 2. Material and Methods

### 2.1. Titanium Disc Samples

Sterile titanium discs (7.5 mm diameter × 2.0 mm thickness, ASTM-F67-95 Grade 4 pure titanium; Southern Implants, Irene, South Africa) were used. The discs had standardized enhanced moderately roughened surfaces (Sa = 1.43 nm) with the same surface topography and chemistry as those of Southern Implants. A total of 55 discs were used; Table 1 indicates how the discs were assigned to the different treatment groups.

### 2.2. Biofilm Formation

Frozen (−80°C) stocks of* Streptococcus mutans* UAB159 (University of Otago, Dunedin, New Zealand) were streaked on blood agar plates using aseptic technique. These plates were incubated anaerobically at 37°C for 24 h. A single colony of* S. mutans* was cultivated anaerobically at 37°C in a sterile tube containing 10 mL of sterile Brain Heart Infusion (Bacto Brain Heart Infusion, [BHI], Becton, Dickinson and Company, Sparks, USA). After 24 h, 200 *µ*L portions of the culture were transferred to 14 vials each containing 10 mL of sterile BHI broth plus 0.5% sucrose. Two sterile titanium discs were added to each of these vials. The vials were then incubated anaerobically at 37°C for 72 h to allow biofilm formation on the discs. The medium was replaced daily by the aspiration of spent medium from vials and the addition of fresh medium consisting of 10 mL of sterile BHI broth containing 0.5% sucrose. Following medium replacement, vials were returned to the anaerobic incubator. The dense, multilayered biofilm formed after 72 h allowed evaluation of the efficacy of the air-polishing therapy on biofilm removal [19].

### 2.3. Group Allocation and Treatment Protocol

Discs were allocated into two groups: incubated with* S. mutans* (biofilm) or no biofilm. Then, discs of each group were further divided into either no air-polishing or the air-polishing group (Table 1). All treatment (air-polishing) groups were instrumented with AIR-N-GO PERIO device (Satelec Acteon Group, Bordeaux, France) using glycine powder (PERIO AIR-N-GO Powder, Satelec Acteon Group, Bordeaux, France), which has a particle size of 25 *μ*m. The device was used at a pressure of 5 bar with a water flow rate of 20 mL/min. This setting was chosen based on reports that the instrument efficacy is greater at higher pressure and with increased water flow rate [16]. According to previous studies, the distance and the angulation of the device seem to have less influence on the instrument efficacy [20, 21]. Hence, the distance between the tip of the device and the disc was set at 5 mm, at an angle of 90° to the disc for consistency and the operator's convenience. A stent specially designed to hold the device was used to control and standardize the distance, position, and angulation during instrumentation. Air-polishing was performed for 5 s in accordance with clinical reports and the manufacturer's recommendation [17, 22–25]. Following the instrumentation, discs were rinsed for 20 s using an air-water spray (triplex) ejecting sterile distilled water with air at 50 psi to remove potential powder deposits. The distance from the tip of the triplex syringe to the disc surface was 2 cm. Discs that did not receive any air-polishing treatment served as the controls. The same operator performed all procedures on the same dental chair.

### 2.4. Surface Topography, Chemistry, and Roughness

A scanning electron microscope (SEM; JEOL 6700 Field Emission SEM, Tokyo, Japan) was used to capture images of the disc surface before and after the air-polishing treatment. These images were used to examine any alterations to the titanium surface. Electron-dispersive spectroscopy (EDS) analysis was also carried out before and after the treatment to evaluate the surface chemical composition. Backscatter images were taken for the analysis of elemental compositional differences across the disc surface. For SEM and EDS analyses, images were captured at 3, 6, 9, and 12 o'clock positions, at a distance of 300 *µ*m from the centre of the disc. Three magnifications of micrographs were used: 500x, 1500x, and 5000x.

To determine any changes in surface roughness of the titanium discs, a confocal laser scanning microscope (CLSM; Zeiss LSM 710, Germany) was used. Images were captured at 3, 6, 9, and 12 o'clock positions, at a distance of 300 *µ*m from the centre of the disc. One disc of each group was observed. CLSM images were analyzed using ImageJ software (Version 1.47V, National Institutes of Health, Baltimore, USA). The mean roughness parameter *R*_*a*_ was obtained using this software.

### 2.5. Biofilm Quantification

To evaluate the effect of air-polishing on biofilm removal, crystal violet assays were performed. Discs were first washed twice with 500 *µ*L of sterile distilled water. The biofilm was then stained with 500 *µ*L of 0.1% crystal violet (0.1 g of crystal violet stain dissolved in 100 mL of 10% ethanol, stored at room temperature) for 15 min at room temperature. The discs were then washed gently three times with 1 mL of sterile distilled water. The bound dye was solubilized using 500 *µ*L of 100% ethanol and the optical density (OD) of the solubilized dye was measured at 600 nm. The percentage reduction in biofilm caused by the treatment was calculated as follows: ((OD_control_ − OD_Treatment_)/OD_control_) × 100.

### 2.6. Biocompatibility

To evaluate the biocompatibility of the titanium surface, viability testing was undertaken using L929 fibroblast cells. Following the air-polishing treatment, discs were kept in sterile microtitre plate wells until cell viability testing was commenced to avoid microbial contamination. Sterile forceps and sterile gloves were used when handling these discs.

Discs were seeded with L929 fibroblast cells in *α*-MEM at a density of 4 × 10^3^ cells/cm^2^ at 37°C for 30 min. Discs were then incubated in *α*-MEM (containing L-glutamine without antibiotics) supplemented with 10% fetal bovine serum (FBS, Invitrogen, Carlsbad, USA) in 5% CO_2_ in a humidified cell culture incubator (Galaxy mini CO_2_ incubator, New Brunswick Scientific, Enfield, USA) at 37°C for 48 h. The viability of the fibroblasts was measured with a live/dead assay [26, 27]. A CLSM (Zeiss LSM 710, Germany) was used to detect the fluorescence of the live and dead cells. For each disc, a total of four images were captured at 3, 6, 9, and 12 o'clock positions, at a distance of 300 *µ*m from the centre. Quantitative analysis of the images was carried out using ImageJ software. Live cells have intracellular esterase activity and the polyanionic dye calcein is retained inside the cells, producing green fluorescence. In dead cells, EthD-1 dye enters through damaged membranes and generates red fluorescence. Live cells can pump out EthD-1 and so show no red fluorescence. Images were separated according to the color channels. The green and red channels were considered separately. The threshold for each image was determined manually and the area of selected threshold was measured with ImageJ software.

### 2.7. Statistical Analysis

For biofilm quantification, the results were analyzed using the Mann-Whitney test, as the data were not normally distributed. For surface roughness and biocompatibility testing results, ANOVA was used to determine whether there was an overall difference across the groups, and then one-way ANOVA was used to identify where the differences were. Fisher's least significant difference (LSD) post hoc test was applied. For all statistical analyses, *α* was set at 0.05 and *P* < 0.05 was considered to be significant.

## 3. Results

### 3.1. Surface Topography

**Figure 1:**
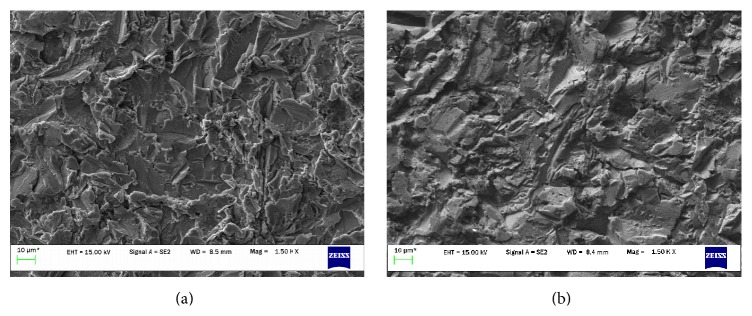
SEM analysis of titanium disc surface morphology: (a) not treated and (b) air-polished. Original magnification: 1500x, scale bar: 10 *μ*m.

The treated discs displayed altered surface morphology when compared by SEM to untreated discs, under all three magnifications. The surface of the treated disc had a smoother profile with less rugosity. Both groups exhibited surfaces that appeared as irregular but well-defined troughs and crests (Figure 1). There were no signs of residual glycine powder on any of the discs.

### 3.2. Surface Chemistry

Titanium, oxygen, and aluminium were present in similar proportions in both treated and untreated discs (Table 2). Both groups had traces (less than 1%) of nitrogen. Amongst the elements considered, titanium was present in the highest amounts followed by oxygen and aluminum. Backscatter images taken under low, medium, and high magnifications did not show any apparent differences in the elemental compositions of the treated and untreated discs.

### 3.3. Surface Roughness

Treated discs had higher mean surface roughness (*R*_*a*_), determined by CLSM, than untreated discs, and this difference was statistically significant (Table 3).

### 3.4. Biofilm Removal

**Figure 2:**
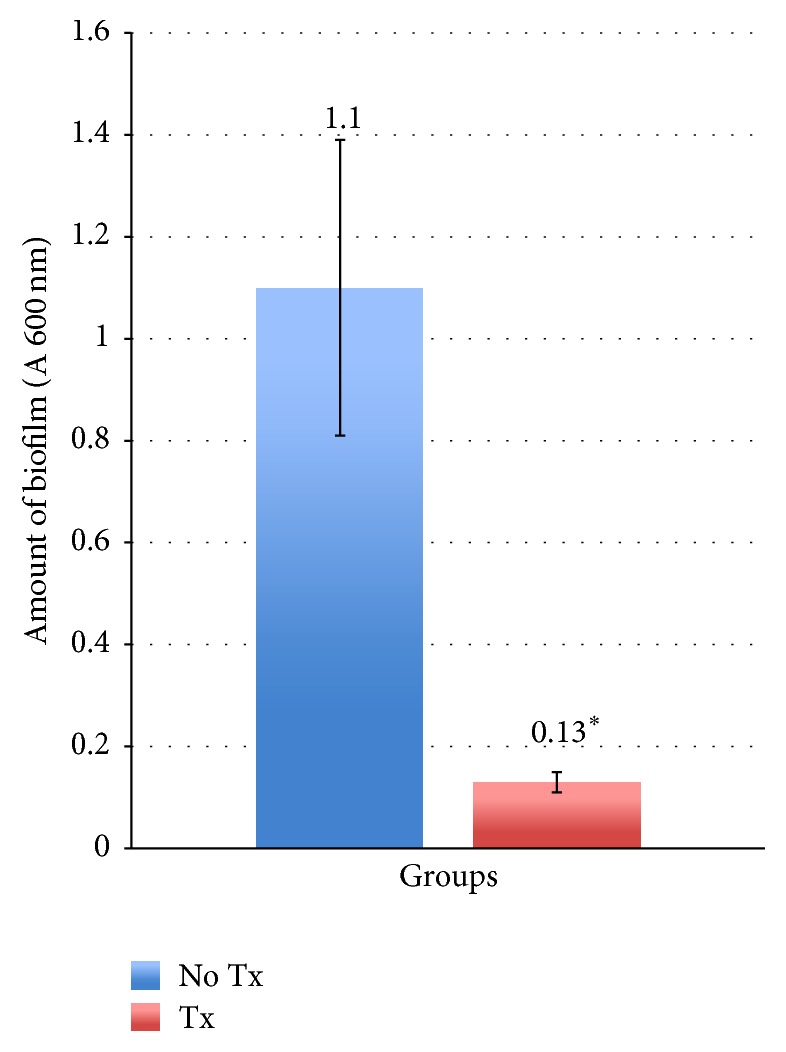
Residual biofilm on titanium discs (*n* = 24). Biofilm mass was quantified by crystal violet staining (absorbance of crystal violet was measured at 600 nm) (^*∗*^ *P* < 0.05).* Note.* No Tx: discs with no air-polish treatment; Tx: discs with air-polish treatment.

The amount of residual biofilm on discs was quantified by the amount of crystal violet dye released from stained biofilms (absorbance measured at 600 nm). The higher the absorbance, the more biofilm was present on the discs. The air-polished discs had significantly lower residual biofilm than untreated discs (Figure 2). The mean amount of residual biofilm in the former group was 8.5 times less than that of the latter group (0.13 ± 0.02 and 1.1 ± 0.29, resp., *P* < 0.05, Mann-Whitney test). This represents an 88% reduction in the amount of biofilm on the air-polished discs.

**Figure 3:**
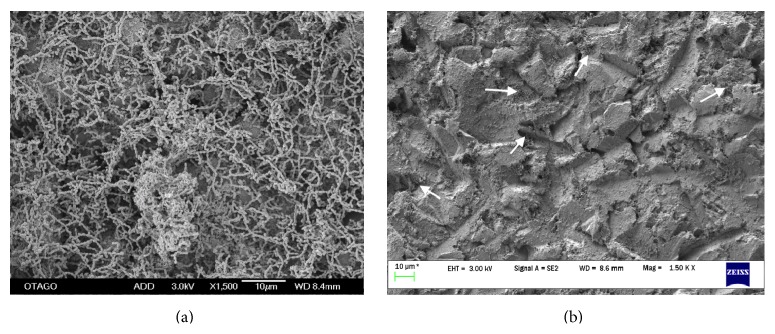
SEM micrographs of biofilms on titanium discs: (a) not treated and (b) air-polished. The white arrows show the presence of polysaccharides and streptococcal remnants after the air-polishing treatment. Original magnification: 1500x, scale bar: 10 *μ*m.

SEM images of treated and untreated discs also revealed changes in the amount of biofilm present (Figure 3). Images of the untreated group, captured under three magnifications (500x, 1500x, and 5000x), showed that the entire disc surface was colonized by a dense network of multiple layers of streptococcal chains enmeshed in polysaccharide fibrils (Figure 3(a)). In contrast, air-polished discs had no visible streptococcal chains or polysaccharide fibrils under low magnification (Figure 3(b)). However, under medium and high magnifications, a network of polysaccharides and small aggregates of streptococci were visible.

### 3.5. Biocompatibility

The areas of live and dead fibroblast coverage of titanium discs were measured and their proportions are shown in Table 4. The differences between the coverage of treated and the coverage of untreated discs by viable cells (and total cells) were statistically significant. It was found that the total area covered by fibroblast cells (whether they were live or dead) on treated discs was half that of the untreated discs suggesting that the change in roughness reduced cell adhesion. However, both groups had the same cell viability (99.6%); therefore, the air-polishing did not affect the viability of cells colonizing the discs.

**Figure 4:**
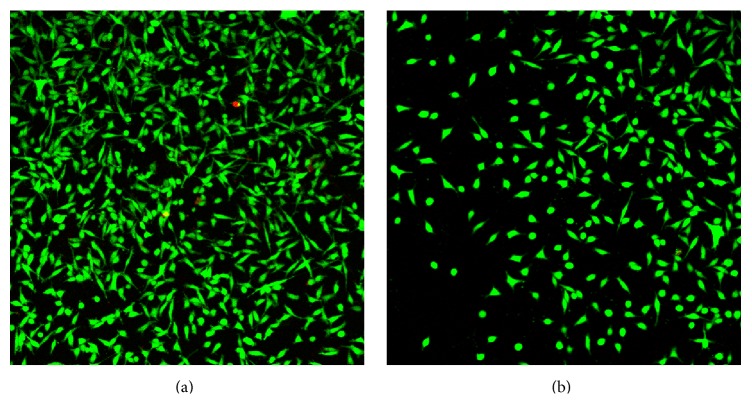
CLSM images of fibroblasts growing on titanium discs: (a) not treated and (b) air-polished. Green cells are live cells, red cells are dead cells, and the black spaces are areas of the discs that are not covered by cells.

The fibroblasts on untreated discs displayed a slightly different morphology compared to those on treated discs (Figure 4). The majority of cells on untreated discs tended to be spindle-shaped or trapezoid-shaped with multiple cytoplasmic extensions, whilst a larger proportion of the cells on air-polished discs tended to be circular, lacking cytoplasmic extensions. These morphological changes indicated that the cells were experiencing stress that may have been brought about by the increased roughness of the air-polished substrate (Figure 4).

## 4. Discussion

Successfully osseointegrated implants can fail if peri-implantitis is left untreated. Peri-implantitis occurs due to accumulation of a biofilm around the implant soft tissues and on implant surfaces, causing an inflammatory response. Once peri-implantitis occurs, it leads to destruction of supporting bone, undermining the stability of the implant [28]. It has been suggested that peri-implant mucositis is a precursor of peri-implantitis [29, 30]. Therefore, early management of peri-implant mucositis by reducing the microbial burden and achieving stable peri-implant soft tissue health can be a key intervention for the long-term success of implants. This research has demonstrated that a 5 s air-polishing treatment using glycine powder resulted in a significant reduction in biofilm load from the titanium surfaces. This pilot study was carried out on 12 discs with bacteria from a single culture and the significant biofilm reduction obtained warrants confirmation with biological replicates. It provides data that will allow a power analysis for further study The short air-polishing instrumentation was sufficient to reduce the biofilm mass 8.5-fold compared to the untreated control and represents a reduction in bacterial burden of 94%. A similar reduction in* S. mutans* biofilms on titanium by air-polishing was reported by Schmage et al. [31].

Many studies have investigated the colonization of different types of surfaces and materials, including titanium, by early colonizer* S. mutans* [32–36]. The bacteria and their extracellular polysaccharide provide attachment surfaces for late colonizers and play a prominent role in initiating the changes in oral microflora as biofilms develop [37, 38]. Our findings suggest that air-polishing treatment could be effective in the prevention of peri-implant diseases by reducing the early colonizer bacterial load, thereby preventing coaggregation of the late colonizers, which are often associated with peri-implantitis.* S. mutans* is known to have multiple efficient modes of adhesion to various surfaces that enable colonization [37]. In contrast, the Gram negative and anaerobic bacteria that are often associated with peri-implantitis have somewhat less efficient adherence mechanisms [39]. From this, we can speculate that an air-polishing treatment, which is effective at reducing more adherent bacterial species, such as* S. mutans*, may well result in the reduction of less adherent species that are often associated with peri-implantitis.

*S. mutans* can tolerate acidic environments and has the capacity to secrete lactic acid as part of its metabolism causing a drop in the pH of growth media and the oral environment [19]. The titanium used in implants has high corrosion resistance due to a titanium oxide (TiO_2_) film created in oxygen-containing environments. However, in the presence of lactic acid, hydrogen peroxide, and high concentrations of fluoride, TiO_2_ can dissolve, resulting in the release of metallic ions. Metallic ions leaching into surrounding soft tissues can cause inflammatory reactions [36]. Recent studies have demonstrated that* S. mutans* on titanium surfaces can reduce the anticorrosive properties of TiO_2_ [35, 36]. This suggests that, as well as having value in prophylactic treatment, air-polishing therapy would be valuable for the ongoing treatment of peri-implant mucositis and peri-implantitis through a reduction of bacterially induced inflammation.

SEM images showed the presence of polysaccharides and streptococcal remnants after the air-polishing treatment. Although there was a significant reduction in bacterial load which will help prevent an inflammatory response, it is not known whether the remaining bacteria were viable, which may allow recolonization of the implant surface and future clinical problems. Also, it is not known what the effect of the residual biofilm would be on the implant biocompatibility. More research is needed to investigate the extent of bacterial recolonization after the air-polishing instrumentation and the biocompatibility of discs with residual biofilm. AIR-N-GO PERIO is a relatively new device; consequently, data on its area coverage for decontamination is still lacking. Further research using different treatment protocols (e.g., optimum time for instrumentation, distance to the surface of treatment, and angulations of the tips) may result in protocols that remove biofilms more effectively.

Air-polishing treatment did not change elemental composition of the titanium surfaces significantly. Elements that are derived from glycine powders are oxygen, nitrogen, and silicone (present in the commercial glycine preparation). Traces of nitrogen (less than 1%) were found in both treated and untreated discs. A slightly higher percentage of oxygen was found in the untreated group and silicone was not present in either group. As these percentages were only indicative values, statistical analysis could not be carried out. For the purpose of the current study (gaining a broad overview on the effect of air-polishing treatment), EDS analysis was sufficient. If more accurate elemental analysis were the main aim of the research, Laser Ablation Inductively Coupled Plasma Mass Spectrometry (LA-ICP-MS) could have been carried out. LA-ICP-MS enables highly sensitive elemental and isotopic analysis to be performed directly on solid samples [40].

CLSM measurements indicated that the average roughness of the treated discs was greater than that of the untreated discs. To date, some studies have documented that increased surface roughness promotes more bacterial adhesion than smooth surfaces [32, 41–43]. Furthermore, it has been reported that bacterial adhesion is affected by multiple factors such as surface free energy, the physicochemical characteristics of the material [32], and the hydrophobicity of the bacterial cell surface [34]. A recent study suggested that it is the wettability of the material that critically affects the bacterial adhesion rather than the surface roughness [44]. Therefore, further studies investigating the effect of varying the amount of air-polishing on surface roughness and on bacterial adhesion are warranted.

The live/dead assay with mouse fibroblast cell line L929 is widely used in biocompatibility testing [27]. The viability of cells on treated discs was the same as that on untreated discs (99.6%) indicating that air polishing did not affect the biocompatibility of the surface. However, the area of treated disks covered by L929 cells was significantly smaller than that of untreated discs. Differences in fibroblast cell morphology were also noticed. Cochis and colleagues [18] concluded that air-polishing treatment with glycine powder reduced bacterial recolonization for 24 h. The reduced cell coverage of treated discs and the observed abnormal cell morphology therefore may be due to air-polishing resulting in suboptimal surface roughness for fibroblast cell adhesion. Another study by Huang et al. [45] also demonstrated that there is optimal surface roughness for cell adhesion and any changes to this can result in reduced cell adhesion.

To conclude, within the limitations of the present* in vitro* study, our findings indicate that 5 s air-polishing treatment using glycine powder significantly reduced bacterial biofilm load on titanium surfaces, although some bacteria and polysaccharide remained. This reduction in the amount of biofilm accompanied the statistically significant increase in surface roughness of the titanium disc surface. There were no apparent differences in surface chemical and elemental compositions following the treatment. Whilst the air-polishing treatment did not seem to affect fibroblast cell viability, the increased surface roughness appears to have reduced the adhesion and/or proliferation of the cells on the surface. This is an area of concern and warrants further investigation.

## Figures and Tables

**Table 1:** Number of discs allocated to treatment groups.

Group	Disc treatment	Roughness analysis	Topographical and chemical analysis (SEM and/or EDS)	Biofilm quantification	Biocompatibility testing	Total number of discs
Biofilm control	None	NA	2 (SEM only)	12	NA	14
Biofilm, air-polish	Air-polish and rinse	NA	2 (SEM only)	12	NA	14
No biofilm control	None	1	2	NA	6	9
No biofilm, air-polish	Air-polish and rinse	3	3	NA	12	18

NA: not applicable.

**Table 2:** Surface elemental analysis of untreated and air-polished discs, showing mean percentage for each element. ^*∗*^Elements originating from glycine powder; ^*∗∗*^elements originating from the disc; t: trace (below 1%). Ti: titanium; O: oxygen; Al: aluminium; N: nitrogen; Si: silicon.

Group	Ti^*∗∗*^ (%)	O^*∗*/*∗∗*^ (%)	Al^*∗∗*^ (%)	N^*∗*^ (%)	Si^*∗*^ (%)
No treatment	58.5	34.3	11.4	t	0
Treatment	54.8	32.3	13.2	t	0

**Table 3:** Surface roughness (*R*_*a*_) of untreated and air-polished discs. The data are shown as means ± standard deviations (^*∗*^ *P* < 0.05 for treatment compared to no treatment).

Group	*R*_*a*_ (*μ*m)
No treatment	2.00 ± 0.45
Treatment	2.45 ± 0.38^*∗*^

**Table 4:** Biocompatibility of air-polished discs. “Live” refers to the proportion of disc area covered by live cells, and “dead” refers to the proportion of disc area covered by dead cells. “Total” refers to the proportion of disc area covered by live and dead cells. The data are shown as means ± standard deviations (^*∗*^ *P* < 0.05 for treatment compared to no treatment) (*n* = 18).

	No treatment	Treatment
Disc coverage by live cells (%)	41.04 ± 19.54	20.36 ± 9.81^*∗*^
Disc coverage by dead cells (%)	0.15 ± 0.16	0.07 ± 0.13^*∗*^
Total disc coverage (%)	41.19 ± 9.58	20.44 ± 9.84^*∗*^
Cell viability (% live cells)	99.60	99.60
